# A real-life experience with eculizumab and efgartigimod in generalized myasthenia gravis patients

**DOI:** 10.1007/s00415-024-12588-7

**Published:** 2024-07-30

**Authors:** Chiara Pane, Vincenzo Di Stefano, Nunzia Cuomo, Alessio Sarnataro, Claudia Vinciguerra, Liliana Bevilacqua, Filippo Brighina, Nicasio Rini, Giorgia Puorro, Angela Marsili, Matteo Garibaldi, Laura Fionda, Francesco Saccà

**Affiliations:** 1 Neuroscience, Reproductive and Odontostomatological Sciences (NSRO) Department, Federico II University, Naples, Italy; 2 Biomedicine, Neuroscience and Advanced Diagnostic (BIND) Department, University of Palermo, Palermo, Italy; 3 Neurology Unit, Medicine, Surgery and Dentistry Department, University of Salerno, Salerno, Italy; 4 Neuromuscular and Rare Disease Centre, Neurology Unit, Sant’Andrea Hospital, Rome, Italy; 5 Department of Neuroscience, Mental Health and Sensory Organs (NESMOS), Sapienza University of Rome, Rome, Italy; 6 Genesis Department, Università Degli Studi Di Napoli “Federico II University”, Via Pansini, 5, 80131 Naples, Italy

**Keywords:** Real-world evidence, Observational, MG-ADL, QMG, Seronegative, Innovative

## Abstract

**Introduction:**

Eculizumab, a complement active antibody, and efgartigimod, an Fc fragment that blocks neonatal Fc receptor, are both approved to treat generalized myasthenia gravis (gMG) patients. The objective of this study is to describe the clinical response to both treatments in a real-life setting.

**Methods:**

We collected baseline and follow-up clinical data using the Myasthenia Gravis-Activities of Daily Living (MG-ADL), and Quantitative Myasthenia Gravis (QMG). We included 63 patients, 32 treated with eculizumab and 31 with efgartigimod. Of the efgartigimod-treated patients, 22 were anti-acetylcholine receptor antibody-positive (AChR-Ab +) and 9 were AChR-Ab- (3 MuSK-Ab + and 6 seronegative).

**Results:**

Both treatments showed similar efficacy relative to the MG-ADL scale reduction (*p* = 0.237). Efgartigimod had a similar effect on both AChR-Ab + and AChR-Ab- (*p* = 0.280). Eculizumab was superior to efgartigimod relative to the QMG score reduction for the entire dataset (*p* = 0.003) and was more likely to achieve a clinical response at the QMG compared to efgartigimod (OR 1.373; *p* = 0.016). Steroid-sparing effect was higher for eculizumab than efgartigimod ( – 16.7 vs  – 5.2 mg of the baseline daily dose at follow-up; *p* = 0.001). Mean speed of prednisone reduction was  – 13.1 mg of the daily dose for each month of follow-up for eculizumab-treated patients and  – 3.2 for efgartigimod (*p* = 0.001). We found three serious events, all not related to treatment in the investigator’s opinion. One eculizumab-treated patient experienced a severe pneumonia and died despite treatment.

**Conclusions:**

Our study provides evidence that eculizumab and efgartigimod can be used in clinical practice to reduce disability in gMG patients. Eculizumab-treated patients had a higher QMG response and steroid sparing effect. Efgartigimod may offer a more flexible schedule due to its cyclical use, no need for vaccination, and efficacy in AChR-Ab- patients.

**Supplementary Information:**

The online version contains supplementary material available at 10.1007/s00415-024-12588-7.

## Introduction

Generalized myasthenia gravis (gMG) is a rare autoimmune disease that causes debilitating muscle weakness [1]. The optimal treatment goal of gMG is to achieve a complete remission, pharmacological remission, or minimal manifestation status (i.e., asymptomatic or no disease-related functional limitation) with minimal adverse events (AEs).

Standard of care (SoC) treatment consists of symptomatic treatment with pyridostigmine, immunomodulating therapies and thymectomy. Immunomodulation is obtained with the use of corticosteroid (CS) and non-steroidal immunosuppressants (NSISTs). Both treatments are non-specific to the pathological mechanism of gMG and are unsatisfactory for several reasons. The use of CS is associated with a burden of adverse events, both in the short and long term, which limits their use. NSISTs are associated with a long delay between treatment start and measurable effect, which is typically in the 6–12 month range. NSISTs can frequently cause intolerable adverse events (i.e., gastrointestinal intolerability, infections, increase in cancer risk) [2]. Despite symptomatic treatment, and use of CSs and NSISTs, many patients continue to experience symptoms of the disease [3, 4]. Also, many of the remaining patients, may present side effects and comorbidities as the consequence of chronic therapy. This limits their quality of life and makes the benefit to risk ratio of SoC very low. Approximately 15% of gMG patients fall in either one of the two categories and for them life with gMG may become a struggle [2].

Recently, two new treatments have been proposed for the therapy of gMG. Eculizumab is a parenteral monoclonal antibody that inhibits C5 cleavage, an essential component of the complement cascade [5]. Efgartigimod is a human IgG1 antibody fragment that binds to and inhibits the neonatal Fc receptor (FcRn) [6]. They are more selective in their mechanism of action, have a short latency of response, and cause a limited number of side effects and comorbidities compared to SoC [7, 8]. Both molecules can be considered the initiators of two new treatment classes with different mechanisms of action (MoA): C5 inhibitor therapy (C5IT), and anti-FcRns.

In a recent meta-analysis of randomized and placebo-controlled trials of innovative therapies in MG with available efficacy data, we concluded that anti-complement and anti-FcRn treatments proved to be effective in gMG patients while rituximab did not show any significant effect on the MG-ADL score [9]. With the limitations of this meta-analysis, such as the very short evaluation interval of anti-FcRns, efgartigimod and rozanolixizumab showed a greater effect on the QMG score in the studied interval, compared to anti-complement and anti-CD20 treatments. No difference was evident when evaluating the MG-ADL between the two treatment strategies.

Real-world data on the use of eculizumab in the United States demonstrated improvement in outcome measures (MG-ADL and QMG), decreased concomitant treatments (IVIg, prednisone, pyridostigmine) [10], reduced MG crises/exacerbations and related hospitalizations in patients with gMG [11]. Clinical improvement with eculizumab was also observed through a 1-year post-marketing Japanese surveillance study. Results showed a reduction of MG-ADL and QMG score, a high responder rate and consistent reduction of daily oral corticosteroid dose [12].

Real-life experience on the use of efgartigimod (EFG) at an Italian reference center suggests that the treatment can greatly reduce hospitalizations, and the use of immunomodulation treatments such as plasma exchange (Plex) or immunoglobulins in patients with gMG [13]. Real-world data from the United Kingdom with patients with gMG treated with efgartigimod demonstrated a reduction of MG-ADL score of ≥ 2 points during the first cycle in 75% of patients [14]. Similarly, a Japanese study reported and MG-ADL decrease from 10.5 ± 4.3 to 6.9 ± 5.1 after efgartigimod first cycle treatment [15].

To date, there is little knowledge on longer-term effects on the MG-ADL, QMG, clinical deterioration rate, treatment retention and CS sparing ability of the two approaches. The recent advances in developing novel target may increase the number of patients that may potentially benefit from innovative treatments. Selection criteria are currently missing in relation to the treatment approach so that current choice between C5IT and anti-FcRn is solely based on local reimbursement criteria or patients’ choice. The aim of the present study is to describe our real-life experience with eculizumab and efgartigimod and show possible differences between the two treatment approaches.

### Patients and methods

We designed a multicenter, retrospective study, involving four Italian Myasthenia Gravis centers. The study was approved by the local ethics committee (122/2024) at the coordinating center and has been performed in accordance with the ethical standards laid down in the 1964 Declaration of Helsinki and its later amendments. All the centers involved in the study asked for written permission for the use of anonymized personal clinical data for research purposes.

Inclusion criteria were age over 18 years, diagnosis of myasthenia gravis and initiating treatment with eculizumab or efgartigimod as part of clinical practice. Data collection took place from September 2023 to January 2024. We collected data using local databases that served as source data. We shared a common database template with pre-defined criteria for data categorization. All files were merged in one common database by a data manager and further processed for data cleaning and analysis.

We collected demographics, and clinical data: age, gender, disease duration, weight, serological status, Myasthenia Gravis Foundation of America (MGFA) clinical classification, thymectomy, previous pyridostigmine, corticosteroid and NSISTs use, baseline pyridostigmine and corticosteroid use, baseline corticosteroid dose, Myasthenia Gravis-Activities of Daily Living (MG-ADL), Quantitative Myasthenia Gravis (QMG), adverse events, clinical exacerbations, MG crisis, hospitalizations, prednisone and concomitant therapy dose variation during therapy, treatment suspension and reason for suspension.

### Treatment schedule

Under the AIFA scheme, eculizumab was administrated to adult patients with anti-acetylcholine antibodies (AChR-Ab +) gMG showing an MG-ADL score ≥ 6, MGFA ≥ III, that had been treated with corticosteroids and ≥ 2 NSISTs. For the last criterion, an exception was possible for those patients showing a contraindication to corticosteroids and/or NSISTs.

Eculizumab was administered through a loading and maintenance phase. Loading phase consisted of four weekly and consecutive i.v. doses of 900 mg of eculizumab, followed one week later by one dose of i.v. eculizumab 1200. Patients then received 1200 mg i.v. eculizumab every 14 days. Eculizumab was administered through an outpatient setting at the reference hospital for the first five infusions. Afterward, patients could opt for home infusions or continue in the outpatient setting.

Patients treated with eculizumab were vaccinated with a tetravalent and anti-serotype B meningococcal vaccination, with the last being performed at least 14 days before the first eculizumab dose. In case this was not possible, patients received amoxicillin until this occurred.

Efgartigimod was administrated to adult patients with AChR-Ab + and AChR-Ab- gMG showing an MG-ADL score ≥ 5, MGFA ≥ II, that had been treated with corticosteroids and ≥ 1 NSISTs. For the remaining criterion, an exception was possible for those patients showing a contraindication to corticosteroids and/or NSISTs.

Efgartigimod was administrated i.v. at a dose of 10 mg/kg (or 1200 mg for patients weighing ≥ 120 kg) once weekly for 4 consecutive weeks as one treatment cycle. Subsequent cycles were administered based on clinical evaluation using the MG-ADL scale or based on patients’ and physicians’ choice, but only if the MG-ADL increased at least 2 points from the best achievable response after a treatment cycle.

All patients were not treated with eculizumab/efgartigimod soon after rescue therapy, leaving at least 30 days after treatment with IVIg/PLEX.

### Myasthenia gravis-activities of daily living (MG-ADL) [16]

The MG-ADL is an 8-item patient-reported scale to assess MG symptoms and their effects on daily activities. It evaluates the capacity to perform different activities of daily living, such as talking, chewing, swallowing, breathing, brushing the teeth/combing the hair, or arising from the chair, and it also assesses double vision and eyelid droop. It is a discrete quantitative variable in which the 8 items are rated from 0 to 3 and the total score can point from 0 to 24, with higher total scores indicating more impairment. The assessments to be performed using MG-ADL do not require any equipment to assess MG symptoms and their effects on daily activities. The MG-ADL was performed weekly in-person during the IV infusions at the hospital and through phone interviews during the intertreatment period. The MG-ADL was performed in-person at baseline and during the first treatment cycle of efgartigimod and the induction phase of eculizumab. After that, data were obtained through phone interview on a weekly basis, and in-person every time patients reached the treating center, i.e., at every efgartigimod treatment cycle, and every 3 months for eculizumab.

### Quantitative myasthenia gravis (QMG) [17]

The QMG quantifies disease severity based on impairments of body functions and structures as defined by the International Classification of Functioning, Disability and Health (WHO 2001). The QMG consists of 13 items that assess ocular, bulbar, and limb function. Out of the 13 items, 6 are timed tests of endurance measured in seconds. Each item has a possible score from 0 to 3. The total possible score is 39, where higher total scores indicate more severe impairments. It is based on quantitative testing of specific muscle groups to assess limb function. It requires minimal equipment, such as spirometer, mouthpieces that fit the spirometer, nose clips, stopwatch, cups and water for swallowing tests, goniometer, dynamometer, and is based on physician’s examination. The QMG was performed at baseline for all patients. For efgartigimod, we collected QMG data at week 1 and 4 of each treatment cycle. For eculizumab, we collected QMG data at baseline, at the end of the induction cycle (week 5), and every 12 weeks (treatment week 12, 24, 36, 48). Since efgartigimod infusion frequency varied between patients, we compared follow-up QMG for eculizumab to the closest week 4 QMG available for each treatment cycle for efgartigimod patients to previous time points (i.e., 12, 24, 36, 48 weeks). This resulted in a minimally different time point between the two treatments.

### Statistics

A descriptive analysis is provided for baseline variables. This includes frequency and percentage (for categorical variables), or mean and standard deviation (for continuous variables). We compared baseline demographics using an unpaired t test or chi-square test when appropriate. We divided the efgartigimod-treated population in AChR-Ab + and AChR-Ab-. We compared the entire population, or Eculizumab vs AChR-Ab + Efgartigimod-treated patients.

We analyzed the difference between treatments relative to the MG-ADL, QMG, and prednisone dose using a General Linear Model for repeated measures (GLM-RM). Since the Mauchly’s test of sphericity was significant, we used the Greenhouse–Geisser correction. We used the effect of time to estimate the overall reduction of scales after treatment, and the combined effect of time*treatment group to estimate the impact of each treatment. We report the *F*-test value for each comparison.

We defined MG-ADL responders as patients achieving a reduction of at least 2 points relative to baseline, and QMG responders if the reduction was at least 3 points. Responder analysis was conducted using first a Generalized Linear Model (GLM) with a negative binomial distribution with log-link, to estimate the overall significance and to calculate the odds ratio (OR) of one treatment over the other. We then used a logistic regression to estimate the OR at every time point and multiplied it through the general OR obtained at the GLM. For MG crisis/deteriorations, we used a Generalized Estimating Equation (GEE) with a negative binomial model with log-link, to estimate the impact of treatment on clinical events considering the year before treatment start and the observation period during treatment. Since treatment duration was different between each patient, we used the ln of days of follow-up as an offset variable.

We analyzed treatment persistence using a Cox regression model with observation time as the dependent variable, drop from treatment as the defining status, and other variables as covariates.

This was an intention-to-treat analysis and missing data were dealt with the last observed carried forward method, applied to all missing data occurring from baseline to last observation. Missing data were occasional and < 5% of total. Since none of the available covariates had a significant impact on dependent variables, not even at the *p* < 0.1 threshold, we did not account for then for further analysis.

Significance was set at 0.05, two-tailed. Statistical analysis was performed using SPSS version 29.0.1.0 running on MACOS 12.6.6. No statistical power calculation was conducted prior to the study. We compared treatment effects up to 48 weeks from baseline as after that time point the number of followed patients decreased and made it difficult to generalize results.

## Results

We included 63 patients, 32 treated with Eculizumab and 31 with Efgartigimod. Both populations were comparable at baseline except for a higher age for Eculizumab-treated patients (60.6 ± 15.5 vs 51.9 ± 14.4; *p* = 0.025; Table 1). Of the Efgartigimod-treated patients, 22 were AChR-Ab + and 9 were AChR-Ab- (3 Ab-MuSK + and 6 seronegative). Only two patients had been pre-treated with Rituximab (more than six months prior to new treatment start), one AChR-Ab + and one MuSK-Ab + , both treated with Efgartigimod. Two Efgartigimod-treated patients were treatment failures to Ravulizumab, and two Eculizumab-treated patients were treatment failures to Efgartigimod.

**Table 1:** General demographics

Variable	Eculizumab (*n* = 32)	Efgartigimod (*n* = 31)	Total (*n* = 63)	*p*	Efgartigimod AChR-Ab + (*n* = 22)	*p*^#^
Gender (F/M)	18/14	22/9	40/23	0.225	16/6	0.218
AChR-Ab +	32	22*	54	–	22	–
Age	60.6 ± 15.5	51.9 ± 14.4	56.3 ± 15.5	**0.025**	50.9 ± 16.2	**0.032**
Disease duration	10.8 ± 9.4	12.6 ± 9.6	11.7 ± 9.5	0.468	12.2 ± 9.8	0.623
Thymectomy	15	15	30	0.904	15	0.122
Previous Py use	30	30	60	0.573	22	0.232
Previous CS use	30	26	56	0.212	19	0.358
Previous NSIST ≥ 1	23	25	48	0.187^$^	19	0.147^$^
Previous NSIST ≥ 2	11	8	19	-	6	-
Previous NSIST ≥ 3	2	3	5	–	2	–
Previous IVIg	22	20	42	0.722	15	0.965
Previous PLEX	8	9	17	0.718	7	0.583
Follow-up days (median and range)	247.5 (30–720)	253 (28–681)	253 (28–720)	0.238	231 (28–681)	0.221
MGFA II	4	10	15	0.203	8	0.155
MGFA III	23	19	41	–	13	–
MGFA IV	4	2	6	–	1	–
MGFA V	1	0	1	–	0	–
MG-ADL	11.0 ± 3.4	9.9 ± 4.2	10.5 ± 3.8	0.243	9.9 ± 4.5	0.281
QMG	17.8 ± 6.1	15.3 ± 5.2	16.5 ± 5.8	0.092	14.9 ± 4.7	0.068

AChR-Ab+ = anti-acetylcholine receptor antibody positive; Py = Pyridostigmine; CS = Corticosteroids; NSIST = Non-steroidal Immunosuppressant; IVIg = Intravenous Immunoglobulins; PLEX = Plasmapheresis; MGADL = Myasthenia Gravis Activities of Daily Living scale; QMG = Myasthenia Gravis quantitative scale. ^*^6 patients treated with Efgartigimod were seronegative, and 3 were Ab-MuSK+; ^$^p value refers to the distribution of the total number of NSISTs across treatment groups; ^#^p value refers to Eculizumab vs Efgartigimod AChRAb+ only patients

Efgartigimod treatment cycles were administered based on treating physicians’ decision. We found a median interval of 56 days (range 28–120 days) between the first administration of the first Efgartigimod cycle and the first administration of the second cycle. We found similar administration intervals for subsequent cycles (Table 2), and a mean interval for the first 6 cycles of 57 days.

**Table 2:** Efgartigimod treatment cycle interval

Intertreatment distance	Median	Min	Max
II cycle (*n* = 29)	56	28	120
III cycle (*n* = 22)	63	35	152
IV cycle (*n* = 17)	60	49	105
V cycle (*n* = 15)	56	39	133
VI cycle (*n* = 13)	50	35	105

Intertreatment distance is shown as the distance in days between the first Efgartigimod administration of one cycle to the first administration of the next cycle. Cycles after the 6th are not shown for limited number of patients

Both treatments were overall well-tolerated despite adverse events were reported in both groups (Supplementary File). We found three serious events, all not related to treatment in the investigator’s opinion. One Eculizumab-treated patient experienced a severe pneumonia and died despite treatment.

### MG-ADL

**Fig. 1:**
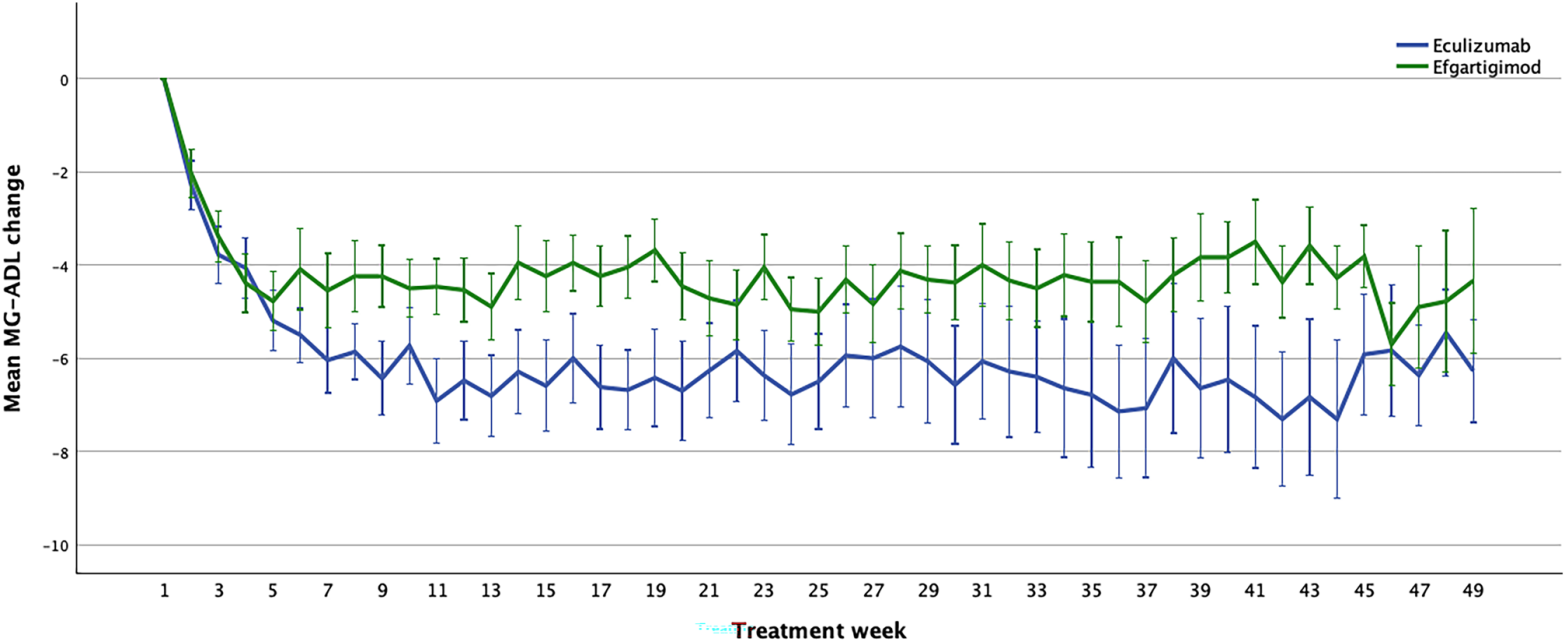
MG-ADL response in Eculizumab and Efgartigimod-treated patients. *MG-ADL* Myasthenia Gravis Activities of Daily Living scale. Error bars show the standard error mean

Treatment with both therapies significantly reduced the MG-ADL score (F = 5.1; *p* < 0.001; Fig. 1). This difference was already significant after one week (F = 18.0; *p* < 0.001) and remained significant after 4 weeks (F = 59.4; *p* < 0.001), 12 weeks (F = 71.3; *p* < 0.001), 24 weeks (F = 102.1; *p* < 0.001), 36 weeks (F = 58.8; *p* < 0001), and 48 weeks of treatment (F = 28.5; *p* < 0.001). Mean differences at significant time points compared to baseline are available in the Supplementary File.

**Fig. 2:**
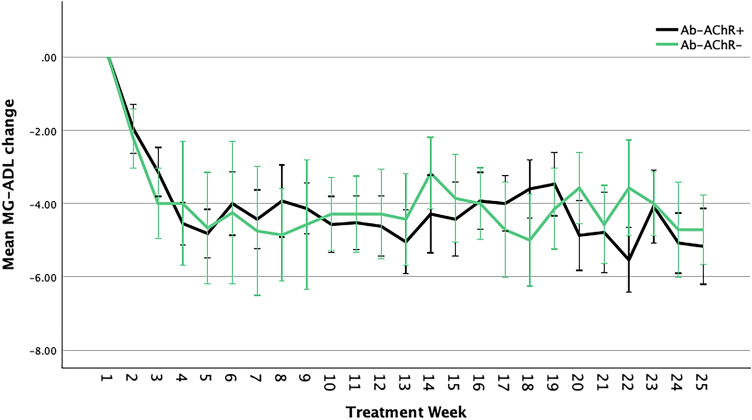
MG-ADL response in Efgartigimod-treated AChR-Ab + and AChR-Ab- patients. *MG-ADL*  Myasthenia Gravis Activities of Daily Living scale, *AChR-Ab +*  anti-acetylcholine receptor antibody positive, *AChR-Ab-* anti-acetylcholine receptor antibody negative. Error bars show the standard error mean

We did not find any significant differences when comparing eculizumab vs efgartigimod-treated patients relative to the MG-ADL score reduction in the observed interval (F = 1.3; *p* = 0.237). Efgartigimod had a similar effect on both AChR-Ab + and AChR-Ab- patients (F = 1.282; *p* = 0.280; Fig. 2).

When considering AChR-Ab + patients only, treatment did not have a significant impact on the MG-ADL reduction in the observed interval (F = 0.961; *p* = 0.457).

**Fig. 3:**
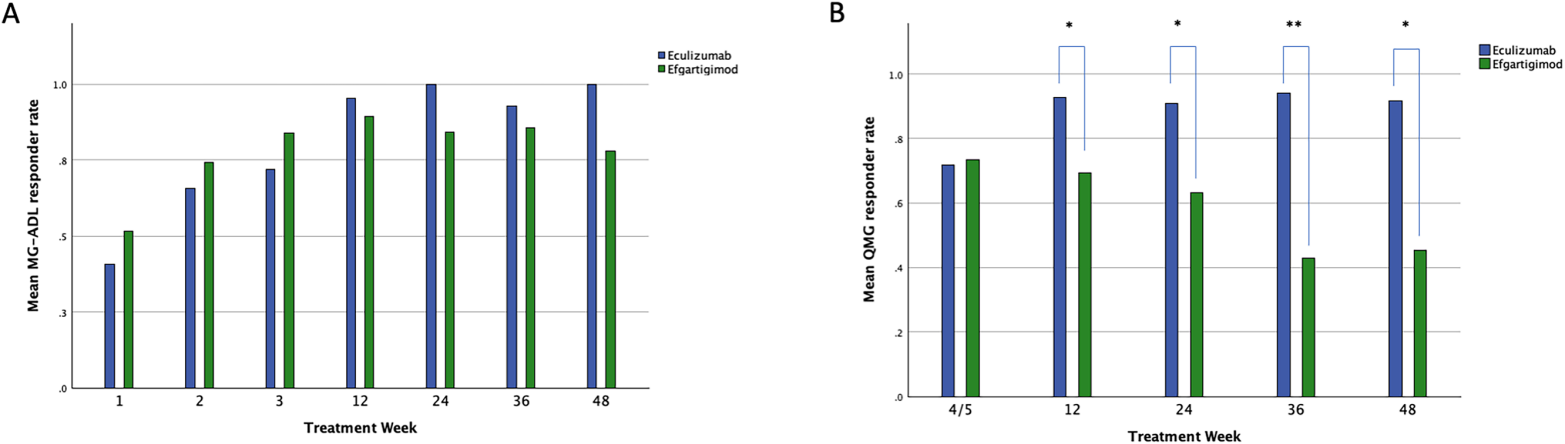
Responder Rate at the MG-ADL and QMG scales. **A** MG-ADL responder rate at different time points. **B** QMG responder rate at different time points. *MG-ADL* Myasthenia Gravis Activities of Daily Living scale, *QMG* Myasthenia Gravis quantitative scale. *p < 0.05; **p < 0.01

We found no differences between Eculizumab and Efgartigimod treatment at the responder rate analysis (OR 0.970, CI 95% 0.814, 1.156; *p* = 0.732; Fig. 3A).

### QMG

**Fig. 4:**
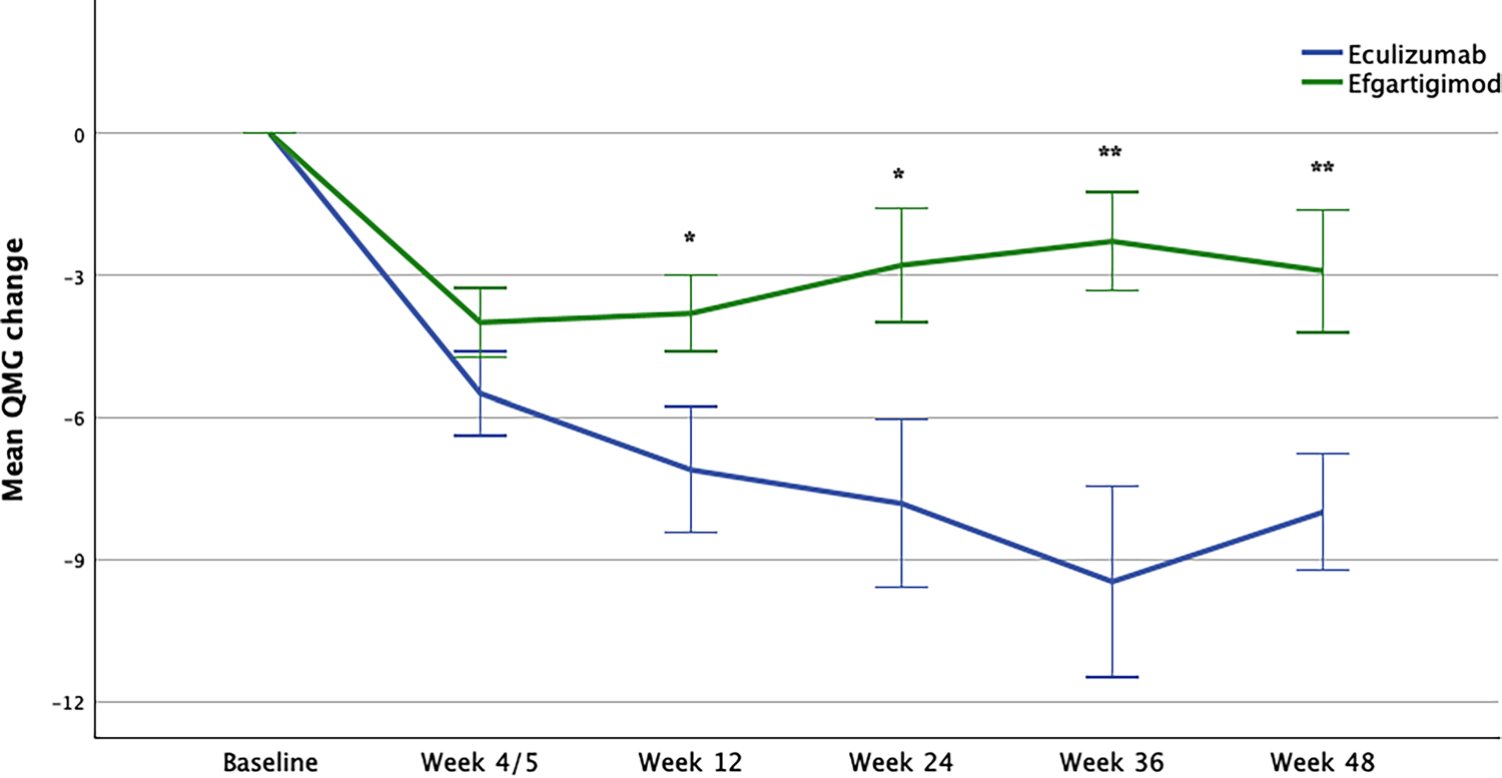
QMG response in Eculizumab and Efgartigimod-treated patients. *QMG* Myasthenia Gravis quantitative scale. *p < 0.05; **p < 0.01. The week 4 interval refers to Efgartigimod-treated patients and week 5 to Eculizumab-treated patients

Treatments significantly reduced the QMG score in the observed interval (F = 17.1; *p* < 0.001; Fig. 4). This difference was already significant after 4 weeks of Efgartigimod and 5 weeks of Eculizumab (F = 33.5; *p* < 0.001) and remained significant after 12 weeks (F = 33.6; *p* < 0.001), 24 weeks (F = 38.3; *p* < 0.001), 36 weeks (F = 27.8; *p* < 0.001), and 48 weeks of treatment (F = 37.1; *p* < 0.001). Mean differences in the QMG compared to baseline for each treatment are available in the Supplementary File.

Eculizumab was superior to Efgartigimod relative to its ability to reduce the QMG score in the studied interval (F = 4.9; *p* = 0.003). This difference was evident when comparing baseline to week 12 (F = 5.3; mean difference  – 3.3; *p* = 0.032), week 24 (F = 6.592; mean difference  – 5.0; *p* = 0.018), week 36 (F = 9.666; mean difference  – 7.2; *p* = 0.005), and week 48 (F = 8.073; mean difference  – 5.1; *p* = 0.010).

For AChR-Ab + patients only, Eculizumab was superior to Efgartigimod relative to its ability to reduce the QMG score in the studied interval (F = 4.4; *p* = 0.008). This difference was evident when comparing baseline to week 12 (F = 5.8; *p* = 0.028), week 24 (F = 7.3; *p* = 0.015), week 36 (F = 6.8; *p* = 0.018), and week 48 (F = 7.145; *p* = 0.016).

Eculizumab was more likely to achieve a clinical response at the QMG compared to Efgartigimod in the studied interval (OR 1.373; CI 95% 1.061, 1.776; *p* = 0.016; Fig. 3B). ORs of individual time points of Eculizumab treatment over Efgartigimod are shown in the Supplementary file.

We found similar results for AChR-Ab + patients only (OR 1.415; CI 95% 1.035, 1.936; *p* = 0.030).

### Deterioration/crisis and treatment discontinuations

**Fig. 5:**
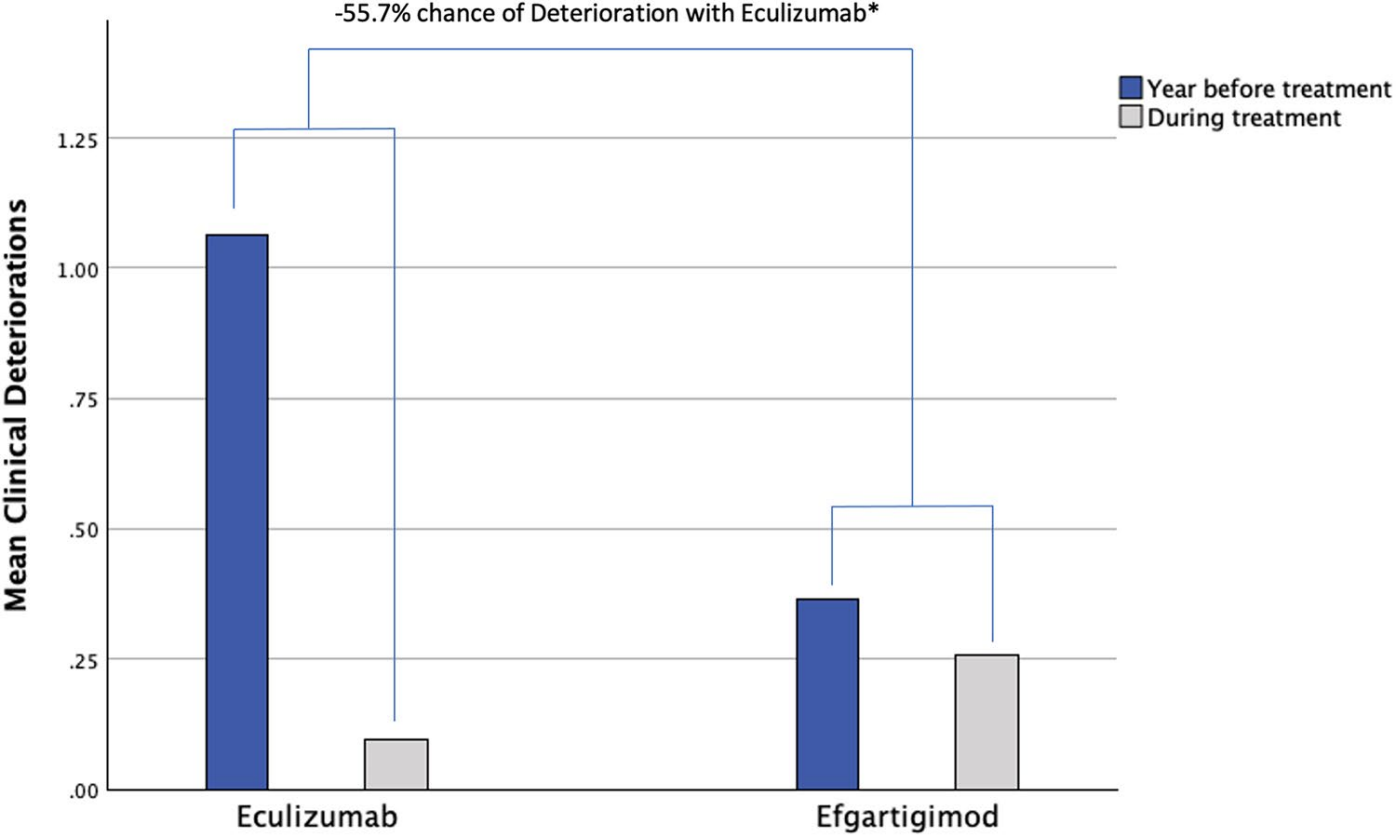
Clinical Events during Eculizumab and Efgartigimod treatment. *QMG* Myasthenia Gravis quantitative scale. *p= 0.043, excluding anti-acetylcholine receptor antibody-negative patients *p* = 0.185

We then compared the number of clinical events (deteriorations, crisis, hospitalizations) the year before treatment start and during treatment. Eculizumab-treated patients showed 1.06 ± 0.4 events during the previous year and 0.09 ± 0.3 during treatment ( – 91.5%). Efgartigimod-treated patients showed 0.37 ± 0.5 events during the previous year and 0.26 ± 0.5 during treatment ( – 29.7%). We found that Eculizumab was associated with 55.7% less chance of having a clinical event during treatment compared to that showed by Efgartigimod treatment (OR 0.543, CI 0.301, 0.981; *p* = 0.043; Fig. 5). Other baseline variables had no impact on clinical events.

We then excluded AChR-Ab- patients and found that patients treated with Efgartigimod had a baseline count of 0.43 ± 0.5 events during the previous year and 0.23 ± 0.4 during treatment ( – 46.5%). The trend was similar to the entire data set, but this time it was not significant (OR 0.675, CI 0.377, 1.207; *p* = 0.185).

**Fig. 6:**
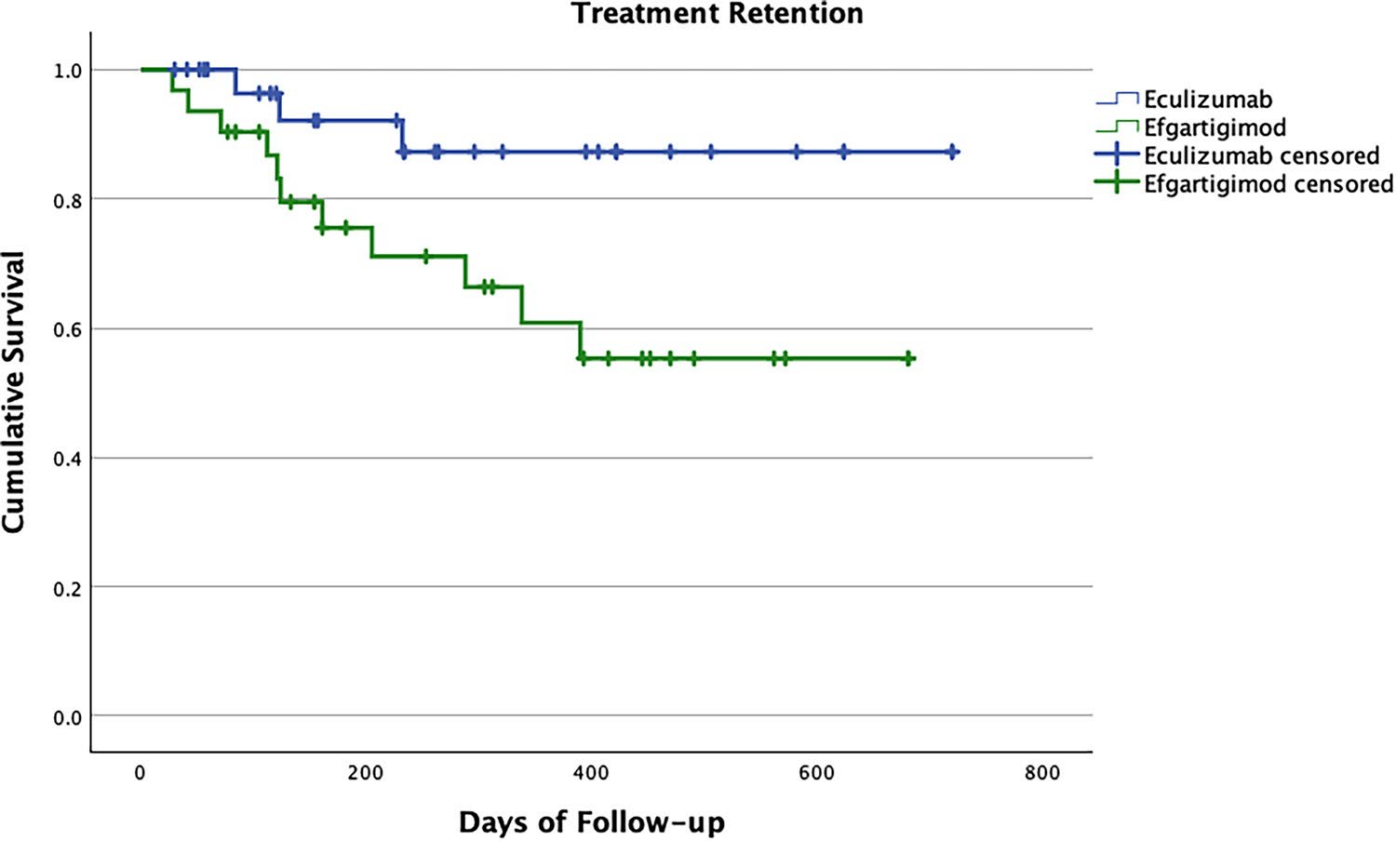
Treatment retention during follow-up with Eculizumab and Efgartigimod

Patients treated with Efgartigimod were more likely to suspend treatment compared to those treated with Eculizumab (HR 3.732; CI 1.041, 13.385; *p* = 0.043; Fig. 6). Main reason for discontinuation was MG deterioration (Supplementary File). Other available factor or covariates did not impact the outcome. We found similar results for the AChR-Ab + group alone (HR 5.126; CI 1.375, 19.115; *p* = 0.015).

### Corticosteroid reduction

**Fig. 7:**
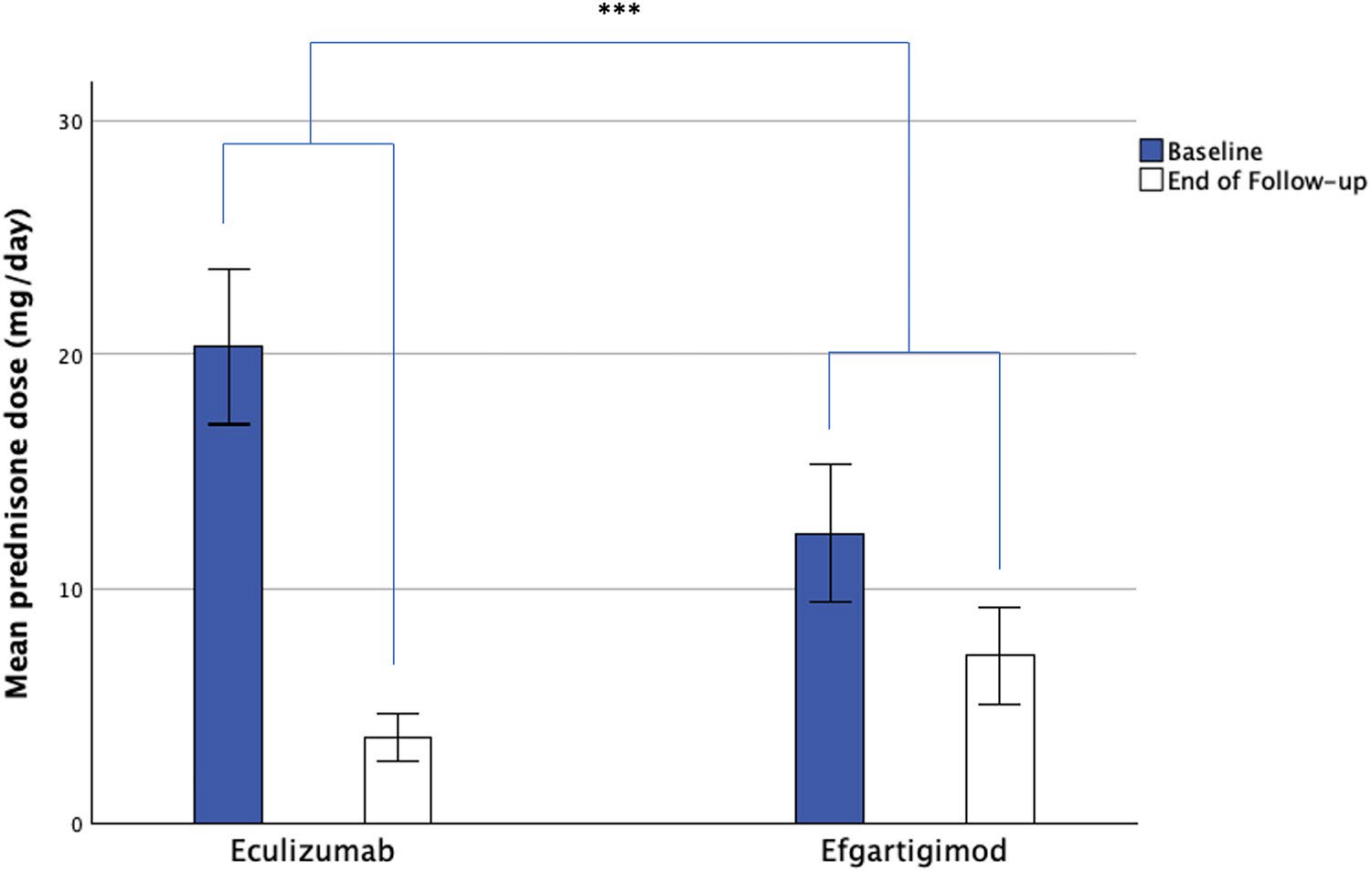
Corticosteroid dose reduction during Eculizumab and Efgartigimod treatment. Treatment effect for all treated patients was in favor of Eculizumab ( – 11.5 mg/day; *F* = 11.728; *p* = 0.001). ***p < 0.001

Baseline prednisone dose was 20.3 ± 18.7 for Eculizumab-treated patients and 12.4 ± 16.4 mg/day for Efgartigimod-treated patients (Fig. 7). At the end of the follow-up period, mean dose was 3.7 ± 5.7 for Eculizumab ( – 16.7, CI  – 10.8,  – 22.5; *p* < 0.001) and 7.1 ± 11.5 mg/day for Efgartigimod ( – 5.2, CI  – 1.9,  – 8.6; *p* = 0.003). Treatment effect for all treated patients was in favor of Eculizumab (F = 11.728; *p* = 0.001). This was also true for the AChR-Ab + population alone (F = 9.035; *p* = 0.004). Percentage drop from baseline values was  – 79.12% for Eculizumab-treated patients, and  – 38.23% for Efgartigimod-treated patients. This does not include two patients treated with Efgartigimod that increased their dose from 0 mg at baseline to 25 and 50 mg/day at the end of follow-up.

Mean speed of prednisone reduction was  – 13.1 mg of the daily dose for each month of follow-up for Eculizumab and  – 3.2 for Efgartigimod (*p* = 0.001). In the AChR-Ab + population, prednisone reduction speed was 4.2 for the Efgartigimod group and still different compared to the Eculizumab-treated group (*p* = 0.040).

## Discussion

Our study is the first real-world report comparing the efficacy of two novel therapeutic approaches in gMG. Patients were treated with Eculizumab and Efgartigimod following clinical practice in four Italian MG reference centers.

Both treatments proved to effectively reduce the MG-ADL score from baseline values. The reduction was evident after just one week of treatment and remained significant for the entire duration of the observation period. In the Efgartigimod-treated cohort, we found similar results when considering AChR-Ab + alone or combined with AChR-Ab- patients. This offers AChR-Ab- gMG patients excellent treatment opportunities with Efgartigimod and promising opportunity with future anti-FcRn treatments, including Rozanolixizumab that is already approved for MuSK-Ab + patients.

The absolute reduction in the MG-ADL was higher than previously reported for eculizumab in the REGAIN trial ( – 7.0 vs  – 4.2) or in the Japanese post-marketing study ( – 5.1), and for efgartigimod in the ADAPT trial ( – 5.0 vs  – 4.5) [5, 6, 12, 18]. It is also striking how the efgartigimod-treated population had a stable response throughout the observation period, despite the administration in treatment cycles vs a continuous scheme for eculizumab. This is a novel finding as this is the first study to report the mean MG-ADL and QMG values of efgartigimod-treated patients in a continuous manner. This was possible, thanks to weekly administration of the MG-ADL and repeated QMG administration, following the recent expert consensus recommendations for the follow-up of gMG patients [19]. The ADAPT/ADAPT + trial reports are missing this data, as are other real-world reports [6, 13, 14, 18, 20, 21]. The reason for a stable response to efgartigimod could be due to an accurate monitoring of patients and to a re-treatment strategy triggered by minimal worsening. This is documented by the low treatment interval between treatment cycles with a mean distance of 57 days. On a yearly basis, this would require 6.5 cycles and 26 i.v. infusions of efgartigimod, identical to the infusion frequency of eculizumab. This administration frequency is different from data reported in the ADAPT + trial of efgartigimod that showed a mean of 4.7 cycles/year [18]. A more frequent administration scheme offers patients the opportunity for a constant disease control. We could speculate that longer intertreatment intervals with efgartigimod could lead to higher fluctuations in the mean MG-ADL values, although offering patients less frequent infusions and a more comfortable schedule.

Both treatments significantly improved the QMG score after 4/5 weeks. After 12, 24, 36, and 48 weeks of treatment, improvement with efgartigimod plateaued, whereas a deeper improvement was evident for eculizumab-treated patients. Absolute improvement for eculizumab-treated patients was higher than previously reported in the REGAIN trial ( – 9.5 vs 4.7). Comparison with efgartigimod trials is difficult as results have been reported as single individual cycles. One additional limitation consists of the different baseline score. Eculizumab-treated patients had higher QMG scores, but the impact of this on treatment response remains unknown. One could speculate that patients with higher baseline scores might have an advantage when looking at improvements after treatment, as there is more room for improvement. On the other side, patients with higher QMG scores have a higher impact of MG on their muscular strength and this could be secondary to a worst NMJ pathology. In this scenario, they would be more difficult to treat patients, and this would support the better effect seen with Eculizumab.

More patients responded to efgartigimod as measured by MG-ADL/QMG scales during the first weeks of treatment as compared to Eculizumab, i.e., during the first treatment cycle (Fig. 3). Although this difference was not significant, the direction of the trend was inverted during week 12/36/48 of follow-up in a non-significant way for the MG-ADL scores and statistically significant for the QMG scores. These results, together with the absolute variation of scales, suggest that lowering pathogenic antibodies may have a short-term advantage on the NMJ function, whereas complement inhibition may be a long-term strategy, and may help preserve a correct anatomical structure of the NMJ. To test this hypothesis further, we should design different observation protocols with more frequent QMG assessments during the first weeks. However, C5IT has recently demonstrated its effectiveness when started during an acute gMG crisis, in patients’ refractory to rescue therapies, but this use remains unclear and deserves future studies [22].

Frangiamore et al. recently reported a cohort of 19 Efgartigimod-treated patients, of which 9 were AChR-Ab + [13]. They showed a reduction of  – 4.5 points at the MG-ADL and  – 3.5 at the QMG after 4 weeks of treatment of the first treatment cycle, and  – 5.6 and  – 4.2 after 4 weeks of the second treatment cycle. This is in line with our findings of  – 4.8 at the MG-ADL and  – 4.0 at the QMG in the same interval of the first treatment cycle (supplementary file). Similarly, Katyal et al. reported a 3.7 point reduction at the MG-ADL and 2.5 point reduction at the QMG after the first Efgartigimod cycle [21]. One study on 17 patients reported a 5.5 point improvement at the MG-ADL after 3 months and 7.1 after 6 months [20].

We would like to highlight how multiple study cohorts of gMG patients have consistent findings of higher response on MG-ADL (subjective scale) vs the QMG (objective scale) while treated with efgartigimod. This is different compared to our finding in eculizumab-treated patients where improvement in QMG score was numerically higher than the MG-ADL. This finding should be confirmed in larger cohorts as there is no clear explanation for this, but it could be due to the different MoA and administration schemes of both treatments.

Both treatments significantly reduced the combined number of MG crisis, deteriorations, hospitalizations, and need for rescue medications. Eculizumab had a significantly higher effect in reducing the total number of events when compared to Efgartigimod (-92% vs -30%), but this was not significant when comparing Eculizumab with Efgartigimod-treated AChR-Ab + population alone ( – 92% vs  – 47%). We think that the latter is a fairer comparison as seronegative patients have been shown to be difficult to treat patients. Also, patients with a higher event rate were selected for Eculizumab treatment causing an imbalance at baseline. Nevertheless, treatment completely inverted this trend leading to an event suppression in the Eculizumab-treated group. Larger studies comparing only AChR-Ab + patients, as well as cohorts with similar baseline event rate, are warranted. A recent study reported one event in 19 Efgartigimod-treated patients during a one-year observation period [13]. Unfortunately, it is difficult to estimate the patient unique event rate of hospitalizations/ICU admissions and the reported use of IVIg/PLEX could be a chronic use and not a rescue medication. Differences in the two cohorts may have impacted on the results, as our patients had higher MG-ADL/QMG scores, despite similar event rate.

Treatment retention has never been thoroughly explored in previous reports. We found that approximately 50% of Efgartigimod-treated patients and 80% of Eculizumab-treated patients were still on treatment at the end of our follow-up. Singer et al. reported 30% discontinuations of Efgartigimod after 5 months of therapy [20]. MG crisis, deterioration and lack of response seem to be the leading cause for discontinuations for both treatments. This may seem disappointing, but it provides additional evidence that innovative treatments are safe and well-tolerated.

Corticosteroid reduction is a key feature of innovative therapies and both Eculizumab and Efgartigimod allowed for a significant reduction of baseline doses. Our study found an Efgartigimod steroid-sparing effect of  – 42% of baseline dose, which is similar to the previously reported value of  – 33% despite a different baseline mean dose [13]. Chronic treatment with Eculizumab was superior in its steroid sparing effect, probably because of its higher disease-modifying effect through its ability in preserving the anatomical integrity of the NMJ. Also, the cyclical nature of efgartigimod treatment, and the use of MG-ADL worsening as a trigger for re-treatment, may have contributed to the slower and less efficient steroid tapering. Future re-treatment strategies and shorted treatment intervals could improve the steroid sparing ability of efgartigimod.

Our study provides evidence that Eculizumab and Efgartigimod can be used in clinical practice to reduce the impact of the disease in difficult to treat MG patients. Eculizumab-treated patients had a higher QMG response and steroid-sparing, higher retention rates, and a trend toward better clinical event prevention, as expected with a treatment intended for chronic use. Efgartigimod may offer a higher response rate during the first weeks of treatment and a more flexible schedule due to its cyclical use.

When setting up the analysis plan, we found ourselves facing the challenge of comparing continuous vs cyclical therapy. There is currently no pre-defined approach as this is a new chapter in how to compare treatments in gMG. The main difficulty we faced was how to compare the QMG scale for both treated populations. We thought that a fair comparison would have been to find the 4th week measurement of an efgartigimod treatment cycle that was nearest to fixed time point for eculizumab (i.e., baseline, 5, 12, 24, 36, and 48 weeks after treatment start). This may of course cause misalignment in QMG measurements, but we think this represents a marginal bias when comparing treatments with sufficient follow-up and frequent treatment cycles. Also, QMG assessments were performed every 12 weeks for eculizumab-treated patients, and at every 1st and 4th week of every efgartigimod treatment cycle, making different comparisons impossible.

Many of the findings presented in this study are similar to previous RWE individual reports, but no study has ever compared different treatment approaches. Limitation of this study lies in the small number of total patients and in the non-randomized treatment allocation. This resulted in Eculizumab-treated patients having higher MG-ADL, QMG, clinical events and baseline steroid dose use, although in a non-significant at statistical tests. We would suggest future studies to compare larger cohorts and possibly match them through propensity score. Alternatively, pragmatic studies with randomized treatment group allocation could be used to eliminate baseline confounders. Considering all these limitations, we recommend caution in interpreting the results of this study, as it is the first a real-life follow-up and comparison of two novel approaches in gMG, namely cyclical anti-FcRn and continuous C5IT.

## Supplementary Information

Below is the link to the electronic supplementary material.

Supplementary material 1 (DOCX 21 kb)
