# Correction: A real-life experience with eculizumab and efgartigimod in generalized myasthenia gravis patients

**DOI:** 10.1007/s00415-025-13061-9

**Published:** 2025-04-29

**Authors:** Chiara Pane, Vincenzo Di Stefano, Nunzia Cuomo, Alessio Sarnataro, Claudia Vinciguerra, Liliana Bevilacqua, Filippo Brighina, Nicasio Rini, Giorgia Puorro, Angela Marsili, Matteo Garibaldi, Laura Fionda, Francesco Saccà

**Affiliations:** 1 Neuroscience, Reproductive and Odontostomatological Sciences (NSRO) Department, Federico II University, Naples, Italy; 2 Biomedicine, Neuroscience and Advanced Diagnostic (BIND) Department, University of Palermo, Palermo, Italy; 3 Neurology Unit, Medicine, Surgery and Dentistry Department, University of Salerno, Salerno, Italy; 4 Neuromuscular and Rare Disease Centre, Neurology Unit, Sant’Andrea Hospital, Rome, Italy; 5 Department of Neuroscience, Mental Health and Sensory Organs (NESMOS), Sapienza University of Rome, Rome, Italy; 6 Genesis Department, Università Degli Studi Di Napoli “Federico II University”, Via Pansini, 5, 80131 Naples, Italy

**Correction: Journal of Neurology (2024) 271:6209–6219** 10.1007/s00415-024-12588-7

In the original version of this article, tables 1 and 2 footnotes were inadvertently omitted.

Table 1 footnote should have read

AChR-Ab+ = anti-acetylcholine receptor antibody positive; Py = Pyridostigmine; CS = Corticosteroids; NSIST = Non-steroidal Immunosuppressant; IVIg = Intravenous Immunoglobulins; PLEX = Plasmapheresis; MGADL = Myasthenia Gravis Activities of Daily Living scale; QMG = Myasthenia Gravis quantitative scale. ^*^6 patients treated with Efgartigimod were seronegative, and 3 were Ab-MuSK+; ^$^p value refers to the distribution of the total number of NSISTs across treatment groups; ^#^p value refers to Eculizumab vs Efgartigimod AChRAb+ only patients

Table 2 footnote should have read

Intertreatment distance is shown as the distance in days between the first Efgartigimod administration of one cycle to the first administration of the next cycle. Cycles after the 6th are not shown for limited number of patients

The original article has been corrected.

