# Supplementary material for: A real-life experience with eculizumab and efgartigimod in generalized myasthenia gravis patients
Source: J Neurol. 2024 Jul 30;271(9):6209–19. doi: 10.1007/s00415-024-12588-7 (PMC11377599; doi:10.1007/s00415-024-12588-7)
Supplement: Supplementary file 1 — Supplementary material 1 (DOCX 21 kb) [file 415_2024_12588_MOESM1_ESM.docx]

**Adverse Events**

| **Adverse Event** | **Seriousness** | **Underlying treatment** | **Treatment related** | **Output** | **Treatment decision** |
| --- | --- | --- | --- | --- | --- |
| Thrombocytopenia | Yes | Eculizumab | No | Resolved | Ongoing |
| Herpes Zoster | No | Eculizumab | No | Resolved | Ongoing |
| Pneumonia | Yes | Eculizumab | No | Not resolved, death | Suspended |
| Metastatic Lung cancer | Yes | Eculizumab | No | Ongoing | Suspended |
| Skin allergic reaction | No | Efgartigimod | Yes | Resolved | Suspended |
| Osteomyelitis | Yes | Efgartigimod | No | Resolved | Ongoing |
| Pneumonia | No | Efgartigimod | No | Resolved | Suspended |
| Urticaria and Angioedema | No | Efgartigimod | Yes | Resolved | Suspended |

**Reasons for Discontinuation**

| **Treatment** | **Reason** |
| --- | --- |
| Eculizumab | Pneumonia |
| Eculizumab | Patient choice |
| Eculizumab | Carcinoma/patient choice |
| Efgartigimod | Clinical Deterioration |
| Efgartigimod | Clinical Deterioration |
| Efgartigimod | Clinical Deterioration |
| Efgartigimod | Clinical Deterioration |
| Efgartigimod | Clinical Deterioration |
| Efgartigimod | Clinical Deterioration/infection |
| Efgartigimod | Patient choice |
| Efgartigimod | Patient choice |
| Efgartigimod | Patient choice |
| Efgartigimod | Adverse event |
| Efgartigimod | Adverse event |

**MG-ADL absolute change from baseline - Eculizumab**

| **Comparison** | **Mean difference** | **95% CI** | **t** | **P value** |
| --- | --- | --- | --- | --- |
| **Baseline vs Week 1** | -2.3 | -1.2, -3.4 | 4.353 | <0.001 |
| **Baseline vs Week 2** | -3.8 | -2.5, -5.0 | 6.213 | <0.001 |
| **Baseline vs Week 3** | -4.0 | -2.7, -5.4 | 6.244 | <0.001 |
| **Baseline vs Week 4** | -5.2 | -3.9, -6.5 | 7.991 | <0.001 |
| **Baseline vs Week 5** | -5.5 | -4.3, -6.7 | 9.251 | <0.001 |
| **Baseline vs Week 12** | -6.8 | -5.0, -8.6 | 7.811 | <0.001 |
| **Baseline vs Week 24** | -6.5 | -4.3, -8.7 | 6.351 | <0.001 |
| **Baseline vs Week 36** | -7.0 | -3.9 -10.3 | 4.753 | <0.001 |
| **Baseline vs Week 48** | -6.3 | -3.8, -8.7 | 5.679 | <0.001 |

| **Comparison** | **Mean difference** | **95% CI** | **t** | **P value** |
| --- | --- | --- | --- | --- |
| **Baseline vs Week 1** | -2.0 | -1.0, -3.1 | 5.465 | <0.001 |
| **Baseline vs Week 2** | -3.4 | -2.3, -4.5 | 5.465 | <0.001 |
| **Baseline vs Week 3** | -4.4 | -3.1, -5.7 | 5.465 | <0.001 |
| **Baseline vs Week 4** | -4.8 | -3.5, -6.1 | 5.465 | <0.001 |
| **Baseline vs Week 5** | -4.1 | -2.3, -5.9 | 5.465 | <0.001 |
| **Baseline vs Week 12** | -4.9 | -3.4, -6.4 | 4.737 | <0.001 |
| **Baseline vs Week 24** | -5.0 | -3.5, -6.5 | 2.318 | <0.001 |
| **Baseline vs Week 36** | -4.8 | -2.9, -6.7 | 2.187 | <0.001 |
| **Baseline vs Week 48** | -4.3 | -0.8, -7.9 | 2.244 | 0.024 |

**MG-ADL absolute change from baseline - Efgartigimod**

| **Comparison** | **Mean difference** | **95% CI** | **t** | **P value** |
| --- | --- | --- | --- | --- |
| **Baseline vs Week 5** | -5.5 | -3.7, -7.3 | 6.195 | <0.001 |
| **Baseline vs Week 12** | -7.1 | -4.4, -9.8 | 5.329 | <0.001 |
| **Baseline vs Week 24** | -7.8 | -4.1, -11.5 | 4.399 | <0.001 |
| **Baseline vs Week 36** | -9.5 | -5.2, -13.8 | 4.683 | <0.001 |
| **Baseline vs Week 48** | -8.0 | -5.3, -10.7 | 6.467 | <0.001 |

**QMG absolute change from baseline - Eculizumab**

| **Comparison** | **Mean difference** | **95% CI** | **t** | **P value** |
| --- | --- | --- | --- | --- |
| **Baseline vs Week 4** | -4.0 | -2.5, -5.5 | 5.465 | <0.001 |
| **Baseline vs Week 12** | -3.8 | -2.2, -5.5 | 4.737 | <0.001 |
| **Baseline vs Week 24** | -2.8 | -0.3, -5.3 | 2.318 | 0.032 |
| **Baseline vs Week 36** | -2.3 | -0.0, -2.2 | 2.187 | 0.048 |
| **Baseline vs Week 48** | -2.9 | -0.0, -2.2 | 2.244 | 0.049 |

**QMG absolute change from baseline - Efgartigimod**

**QMG responder rate (-3 points from baseline) – Eculizumab vs Efgartigimod**

| **Comparison** | **OR** | **P value** | **RR Eculizumab** | **RR Efgartigimod** |
| --- | --- | --- | --- | --- |
| **Baseline vs Week 5** | 0.677 | 0.898 | 71.9 | 73.3 |
| **Baseline vs Week 12** | 7.933 | 0.039 | 92.9 | 69.2 |
| **Baseline vs Week 24** | 8.009 | 0.045 | 90.9 | 63.2 |
| **Baseline vs Week 36** | 29.290 | 0.009 | 94.1 | 42.9 |
| **Baseline vs Week 48** | 18.124 | 0.033 | 91.7 | 45.5 |
